# Supplementary material for: Predicting visual function by interpreting a neuronal wiring diagram
Source: Nature. 2024 Oct 2;634(8032):113–23. doi: 10.1038/s41586-024-07953-5 (PMC11446822; doi:10.1038/s41586-024-07953-5)
Supplement: Supplementary file 6 — CRF and ERF predictions for individual TmY4 and TmY9 cells. Analogous to Supplementary Data 3, but for TmY target types. Shown are the top four monosynaptic pathways, the strongest pathway passing through each of the top ten intermediary types (ranking from Extended Data Fig. 7), and the trisynaptic pathway Tm1–TmY–Dm3–TmY (see the section entitled Prediction of spatial normalization). [file 41586_2024_7953_MOESM6_ESM.zip › DataS4/TmY9q/720575940621480176.pdf]

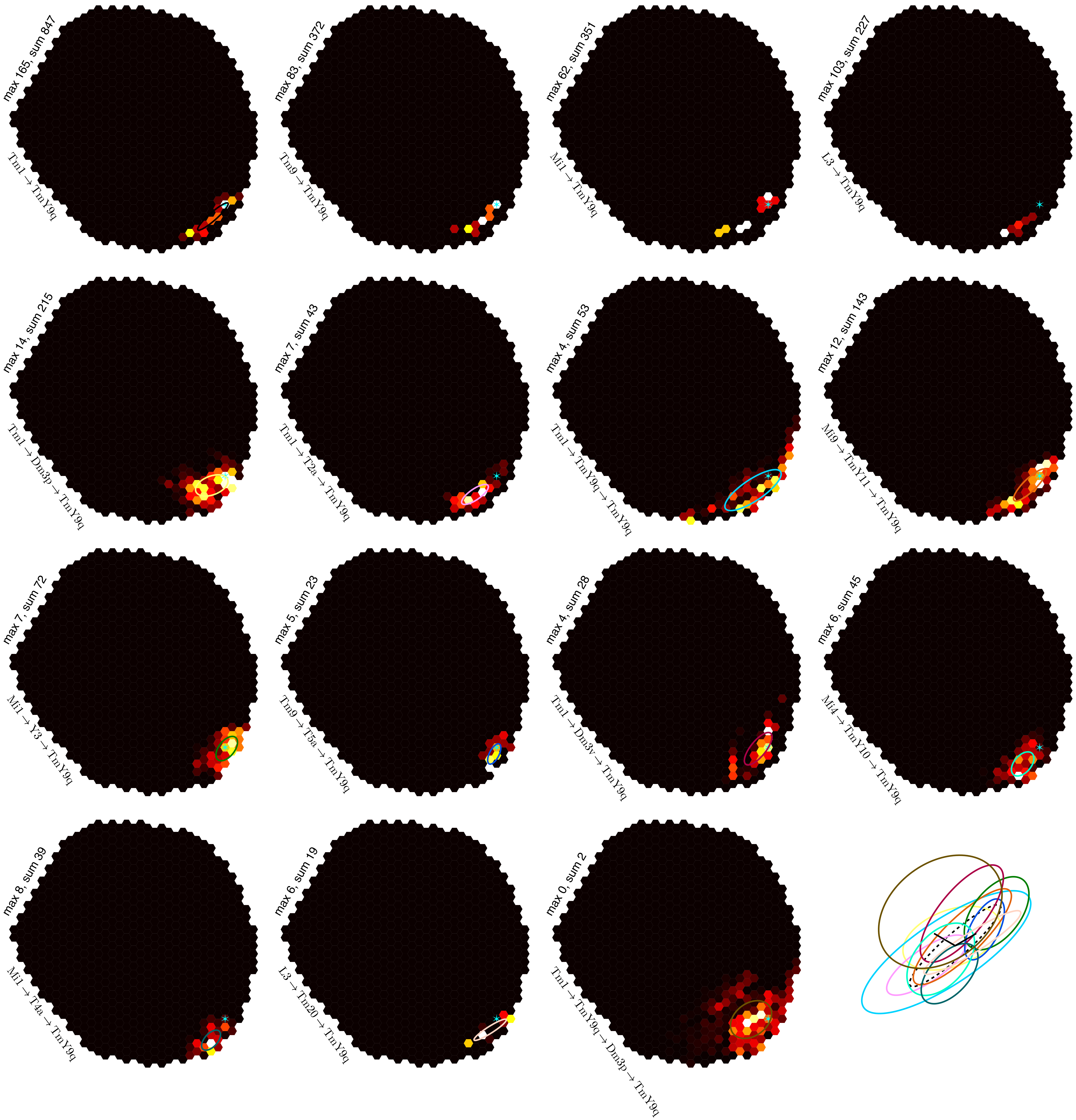
max 165, sum 847
Tm1 → TmY9q
max 83, sum 372
Tm9 → TmY9q
max 62, sum 351
Mi1 → TmY9q
max 103, sum 227
L3 → TmY9q
max 14, sum 215
Tm1 → Dm3p → TmY9q
max 7, sum 43
Tm1 → T2a → TmY9q
max 4, sum 53
Tm1 → TmY9q → TmY9q
max 12, sum 143
Mi9 → TmY11 → TmY9q
max 7, sum 72
Mi1 → Y3 → TmY9q
max 5, sum 23
Tm9 → T5a → TmY9q
max 4, sum 28
Tm1 → Dm3v → TmY9q
max 6, sum 45
Mi4 → TmY10 → TmY9q
max 8, sum 39
Mi1 → T4a → TmY9q
max 6, sum 19
L3 → Tm20 → TmY9q
max 0, sum 2
Tm1 → TmY9q → Dm3p → TmY9q
